# Generalized exfoliative skin rash as an early predictor of supratherapeutic voriconazole trough levels in a leukemic child: A case report 

**DOI:** 10.18502/cmm.6.3.4500

**Published:** 2020-09

**Authors:** Ali Amanati, Parisa Badiee, Mehrzad Lotfi, Ahmad Monabati, Mohammad Ali Faghihi, Majid Yavarian, Nazafarin Hatami Mazinani

**Affiliations:** 1 Professor Alborzi Clinical Microbiology Research Center, Shiraz University of Medical Sciences, Shiraz, Iran; 2 Medical Imaging Research Center, Department of Radiology, Shiraz University of Medical Sciences, Shiraz, Iran; 3 Department of Hematopathology, Molecular Pathology and Cytogenetics, Shiraz University of Medical Sciences, Shiraz, Iran; 4 Center for Therapeutic Innovation, Department of Psychiatry and Behavioral Sciences, University of Miami, Miami, USA; 5 Persian Bayan Gene Research and Training Center, Shiraz, Iran; 6 Hematology Research Center, Shiraz University of Medical Sciences, Shiraz, Iran; 7 Department of Clinical Pharmacy, School of Pharmacy, Shiraz University of Medical Sciences, Shiraz, Iran

**Keywords:** Acute lymphoblastic leukemia, Skin rash, Therapeutic drug monitoring, Voriconazole

## Abstract

**Background and Purpose:**

Skin rashes, mostly seen in children and adolescents, are considered among the most common side effects of azole antifungals. Although therapeutic concentrations of voriconazole (VCZ) have been documented for infected skin, there is no evidence specifying whether specific dermatologic side effects could predict high VCZ serum concentration, especially in high-risk leukemic children.

**Case report:**

Herein, we report a unique skin side effect of VCZ in a 5-year-old boy with T-cell acute lymphoblastic leukemia (ALL) referred to Amir Medical Oncology Center in Shiraz, Iran. The patient experienced erythroderma and macular rashes shortly after VCZ consumption, leading to generalized exfoliative skin rashes. Concurrent to these skin manifestations, VCZ serum concentration reached the supratherapeutic levels despite the recommended VCZ doses. As a result, VCZ was withheld, and the patient was treated with caspofungin. The lesions were resolved gradually within 2 weeks, and the patient successfully completed his treatment course with caspofungin.

**Conclusion:**

The unique case presented in this study emphasizes the need for a high index of suspicion for VCZ toxicity in any patient with atypical dermatologic manifestations, especially generalized exfoliative skin rashes. Based on this report, VCZ supratherapeutic concentration could be predicted early in the course of treatment. Additional therapeutic dose monitoring should be considered to establish a confirmatory diagnosis. It is required to further investigate the toxic effect of high VCZ concentration on the skin epithelium.

## Introduction

Dermatologic reactions are considered among the common side effects of azole antifungals, including voriconazole (VCZ). However, VCZ is accompanied by common dermatologic side

effects for example, fixed drug eruption, skin discoloration, skin photosensitivity [ 1 ], Stevens-Johnson syndrome/ toxic epidermal necrolysis, melanoma, and squamous cell carcinoma [ 2 ]. The VCZ therapy has been reported to be used for the treatment of various types of dermatitis, such as allergic dermatitis [ 3 ], contact dermatitis [ 4 ], and even exfoliative dermatitis [ 5 ]. The pathophysiology of these adverse reactions is different. It may be affected by various risk factors, such as the previous history of liver disease, concurrent hepatic insufficiency, *CYP2C19* polymorphisms, and duration of treatment [ 6 ].

Azoles can accumulate in the skin and skin structures. In this regard, eccrine sweat can transport azoles across the blood-skin barrier, where it binds to keratinocytes and surface lipids [ 7 ]. In addition, azoles can effectively penetrate the skin tissues and accumulate in the stratum corneum. This medicinal group can also persist in the infected skin at therapeutic concentrations 3-4 weeks after treatment [ 8 , 9 ]. Skin rashes usually appear early after treatment, and re- challenge can provoke a similar reaction [ 10 ]. On the other hand, a prolonged use of azoles is associated with skin cancer [ 11 , 12 ].

Despite the previous reports on the association of supratherapeutic VCZ C _trough_ level with some VCZ adverse effects (e.g., VCZ-induced encephalopathy and visual disturbance) [ 13 , 14 ], such an association has not been documented for dermatologic side effects [ 11 ]. Accordingly, it is required to determine the clinical significance of dermatologic manifestations during VCZ treatment, especially in association with VCZ serum concentrations. Regarding this, the present report aimed to raise concern about the emergence of nonspecific skin rashes during VCZ treatment and alarm clinicians to be aware of the potential association between dermatologic side effects and supratherapeutic VCZ C _trough_ level.

## Case report

A 5-year-old boy was referred to the Amir Hospital, an academic medical center affiliated to Shiraz University of Medical Sciences, Shiraz, Iran, on 13 August 2018, due to pallor and prolonged fever. Initial lab testing revealed the white blood count (WBC) of 57,200 cells/µL, hemoglobin (Hb) level of 8.7 g/dl, platelet count of 7,000 cells/µL, C-reactive protein (CRP) of 15 mg/L, and erythrocyte sedimentation rate (ESR) of 47 mm/h. Physical examination revealed cervical lymphadenopathy and mediastinal mass in the first chest radiograph. Accordingly, he was subjected to bone marrow aspiration and biopsy on 15^th^ August. The patient was diagnosed with T-cell acute lymphoblastic leukemia (ALL); as a result, he underwent induction chemotherapy.

Sulfamethoxazole/trimethoprim (2.5 mg/kg/dose of trimethoprim Q12 h/every other day) and ciprofloxacin prophylaxis (10 mg/kg/dose Q12 h) were begun during induction-remission chemotherapy and neutropenic phase, respectively. On 3^rd^ September, after 2 weeks of deep neutropenia (absolute neutrophil count of < 500 cells/µL), he became febrile, while his total WBC count was 180 cells/µL. Sepsis workup was performed, and intravenous piperacillin-tazobactam was started. The blood tests revealed an elevated level of ESR (54 mm/h) and CRP (70 mg/L). Two days later, abdominal pain developed, and a thickened intestinal loop wall was detected in abdominopelvic sonography in favor of neutropenic enterocolitis (typhlitis).

**Figure 1:**
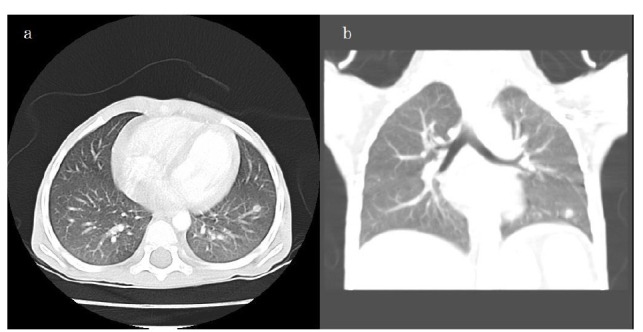
Spiral computed tomography scan of the chest and mediastinum without contrast (with coronal reconstruction) (Multiple axial and coronal images through the chest and mediastinum demonstrate a small nodule measuring about 5×5 mm in the left lower lobe [axial (a) and coronal (b) views]).

Because of continuous fever despite broad- spectrum antibiotics and improvement of sonographic findings, spiral paranasal sinuses and chest computed tomography (CT) scans were requested. Spiral chest CT revealed ground-glass opacity in the left lung (Figure 1). Serum Galactomannan test (GM) became positive with an optical density (OD) index of 0.511 (Platelia™ *Aspergillus* EIA, sera with an index of ≥ 0.50 considered to be positive for GM antigen). The diagnosis of probable pulmonary aspergillosis was established based on the revised EORTC/MSG criteria [ 15 ]. As a result, intravenous VCZ therapy was started from the 8th September (6 mg/kg, every 12 h for one day and then at a dose of 4 mg/kg) [ 16 ].

**Figure 2:**
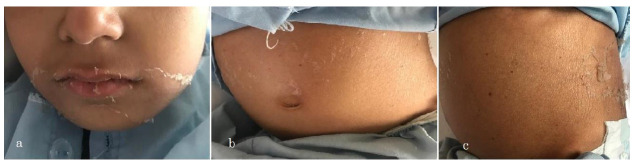
Development of erythroderma and a macular rash within a few days of voriconazole treatment, followed by generalized exfoliation in the face and trunk (a few days later on September 15^th^)

The patient developed erythroderma and macular rash within a few days of VCZ treatment. After the gradual improvement of primary lesion, he developed generalized exfoliation in the face and trunk (Figure 2). The possibility of VCZ toxicity raised, and VCZ dose adjustment (30% dose reduction) was achieved 5 days later. The patient underwent routine VCZ therapeutic dose monitoring (TDM) [ 17 ]. The VCZ C _trough_ levels were much higher than the expected therapeutic range (VCZ therapeutic reference range: 1.0-5.5 mg/L [ 18 , 19 ]) a few days after the onset of treatment. These levels were estimated at 9.56 and 10.98 μg/mL on days 5 and 6, respectively. The VCZ was withheld accordingly on day 8, resulting in the reduction of VCZ C _trough_ levels to subtherapeutic concentration a week later (0.59 and 0.59 μg/mL on 22^th^ and 23^th^ September, respectively).

**Figure 3:**
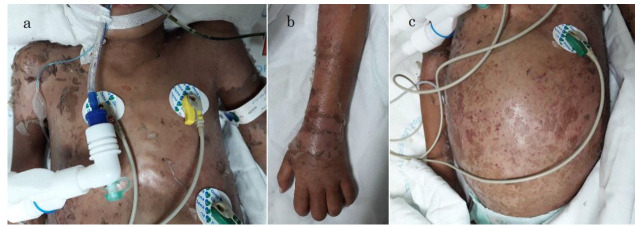
Deterioration of patient condition a week later due to severe neutropenic enterocolitis (typhlitis) (Severe exfoliation occurred on September 21^th^. The patient was transferred to PICU, and mechanical ventilation was started.)

On September 15^th^, the patient's clinical condition deteriorated by the sudden onset of respiratory distress and severe abdominal distention, which necessitated pediatric intensive care unit (PICU) admission. Skin rash progressed in both extremity and trunk (Figure 3). The laboratory test revealed the WBC of 2,200 cells/µL, platelet count of 31,000 cells/µL, ESR of 44 mm/h, CRP level of 146 mg/L, Hb level of 8.5 g/dL, alanine aminotransferase (ALT) of 67 IU/L, and raised aspartate aminotransferase of 25 IU/L.

**Figure 4:**
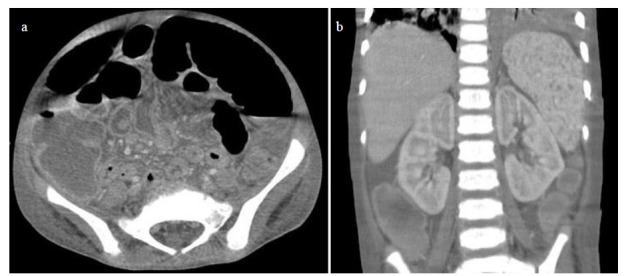
Abdominopelvic spiral computed tomography scan with contrast (with coronal reconstruction) (Multiple axial and coronal images through the abdomen and pelvis after intravenous and oral administration of the contrast demonstrates that diffuse bowel dilatation associated with fluid-filled loops were present down to the distal sigmoid part, in addition to the thickened bowel walls [a]. The liver has a normal homogeneous density with the evidence of periportal edema. In contrast, multiple small splenic lesions [low-density nodular patterns] in addition to inhomogeneous density are observed in the spleen [b]).

Spiral chest and abdominopelvic CT scans were repeated, which revealed collapse consolidation in the left lower lobe and also severe splenic involvement in addition to severe pancolitis (Figure 4). The patient was successfully weaned from mechanical ventilation on 5^th^ October.

**Figure 5:**
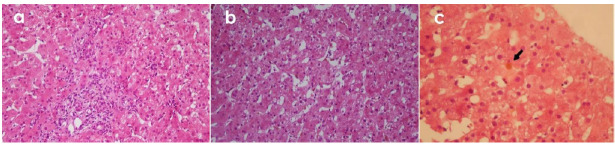
a) Low power view of the liver portal tract and surrounding hepatocytes (The portal tract has slight lymphocytic infiltration with occasional permeation of plates), b) Another portal tract with mild infiltration of lymphocytes without invasion to limiting plates (Hepatocytes are unremarkable.), c) Higher view of hepatocytes without lobular hepatitis (There is a focal area of cholestasis [black arrow] in the center; hematoxylin- eosin X400]).

Liver biopsy was performed due to the detection of multiple hypoechoic lesions in the follow-up abdominal ultrasound examination (Figure 5). Liver biopsy samples subjected to polymerase chain reaction (PCR) were positive for aspergillosis (for two different specimens). The real-time PCR was performed as described previously [ 20 ]. *Aspergillus* flavus was cultured on Sabouraud dextrose agar media (Merck, Germany) on the liver biopsy samples (the mycological study was performed at Professor Alborzi Clinical Microbiology Research Center, Shiraz University of Medical Sciences).

Genomic DNA analysis was performed on cytochrome P450, family 2, subfamily C, polypeptide 19 (*CYP2C19*) enzyme to determine possible allelic dysmorphism (e.g., poor metabolizer status of *CYP2C19*). This gene product contributes to the metabolism of a large number of clinically relevant drugs and drug classes, such as antidepressants, benzodiazepines, mephenytoin, proton pump inhibitors, and antiplatelet clopidogrel as a prodrug. The *CYP2C19* gene haplotype primers were designed by Primer3 software (version 0.4.0). The analysis of haplotype was performed by direct bi-directional sequencing on the ABI 3130XL sequencer. The "Chromas 2.6.6." Software was used for data exploration. The analyzed amplicon was checked for rs12248560 (c.-806 C>T) at the promoter site, rs4986893 (c.636 G>A: cd212), and rs4244285 (c.681 G>A: cd227) polymorphisms (based on NM_000769.2] by bi-directional sequencing. The result was suggestive of a homozygote CC, GG, and GG pattern, which was compatible with homozygote wild-type allele (Allele *1, normal metabolizer).

**Figure 6:**
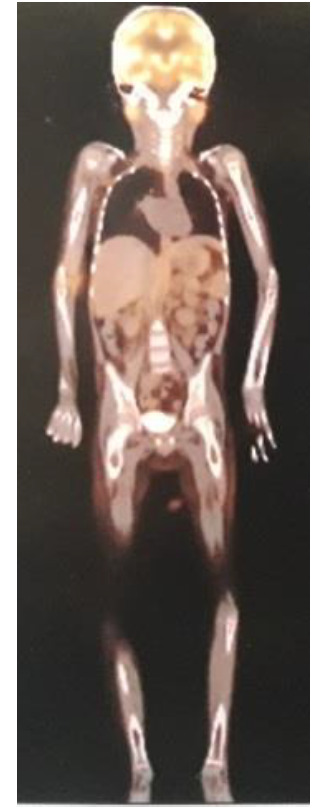
Result of fluorodeoxyglucose positron-emission tomography (FDG/PET) scan after four months of antifungal treatment (Despite primary multiorgan involvement in the lung, liver, and spleen, no evidence of metabolically active lesion was noted throughout the body in the follow-up FDG/PET scan.)

**Figure 7:**
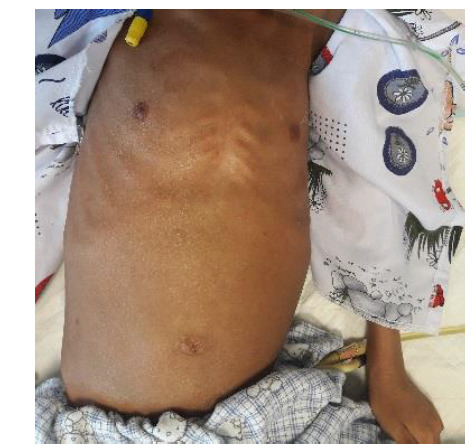
Gradual improvement of patient's rash within a few weeks (All anti-cancer drugs were continued according to the protocol. No skin reaction occurred after the re-use of chemotherapeutic agents.)

The patient received 4 months of caspofungin as a second antifungal choice to complete his treatment course. He was subjected to fluorodeoxyglucose positron emission tomography on February 6, 2019. The results revealed no evidence of metabolically active lesions throughout the body (Figure 6). The patient continued to be on close follow-up without any complaint at the time of writing this report (i.e., almost 28 months after the appearance of the first dermatologic symptoms [Figure 7]).

## Discussion

The primary aim of this study was to highlight the clinical significance of VCZ-TDM in a high-risk population, such as cancer patients. The second aim of this report was to emphasize the correlation of VCZ rash with high VCZ C _trough_ levels. In contrast to the complications of the central nervous system (CNS) [ 21 - 23 ], VCZ C _trough_ levels have no association with skin rash, the severity of liver dysfunction, and visual disturbance [ 11 ]. Researchers have failed to find any correlation between VCZ concentrations and such adverse events [ 24 ].

This unique case report signifies the potential predictor role of VCZ rash as an early predictor of high VCZ C _trough_ levels even when VCZ-TDM is not available. Dermatologic reactions are considered the most common adverse effects of VCZ treatment just after visual disturbance, which can occur in up to 30% of patients [ 25 ]. A photosensitivity reaction has been reported in about 7-12% of the patients who are on VCZ treatment [ 1 , 11 , 26 ]. Mild erythematous eruptions usually occur on sun-exposed areas, such as the face and hands [ 25 ]; however, more severe reactions, including Stevens-Johnson syndrome and toxic epidermal necrolysis, can also take place [ 27 - 29 ]. Consideringthepatient'smedicationhistory (methotrexate (MTX], dexamethasone, Adriamycin, and vincristine), only MTX could cause similar dermatologic side effects. According to its dose and half-life (4 gram/m^2^, maximum half-life=5.8 h, and maximum washout period=29 h), it is inconceivable that MTX had a therapeutic serum level at the time of first skin reactions. On the other hand, only vincristine had the therapeutic serum level at the time of dermatologic manifestations when he developed skin rashes. Vincristine can enhance the VCZ C _trough_ level and vice versa; accordingly, this drug interaction may have led to a high VCZ C _trough_ level in our case.

*CYP2C19* allelic polymorphisms account for another important factor that could be affecting VCZ metabolism and directly modify bioavailability and clearance of VCZ. Currently, 529 various *CYP2C19* diplotypes/phenotypes have been described with different risk priorities. Among these, 55 cases are defined as "poor metabolizers", and an additional 60 cases are regarded as "likely poor metabolizers." The poor metabolizer phenotypes prone VCZ recipients to a greater risk of VCZ toxicity [ 6 ]. The consideration of *CYP2C19* genotyping before VCZ treatment may be helpful in the management of high-risk populations, such as patients with hepatic insufficiency or those receiving medications that may increase the risk of VCZ toxicity by drug-drug interactions (e.g., proton- pump inhibitors) [ 30 ].

While dermatologic side effect is the crucial manifestation of VCZ toxicity, based on histo- pathologic findings, “drug-induced liver injury” also may be considered another potential sign of high VCZ C _trough_ level in our case. The VCZ toxicity could occur in patients with preexisting liver dysfunction that could be noted by an increase in transaminases. The worsening of the liver function tests can observe even after at least four doses of VCZ in patients with preexisting severe liver dysfunction. Notably, this phenomenon occurs in patients who have received a higher initial dose of VCZ [ 31 ].

In this unique case, the lymphocytic infiltration and focal area of cholestasis without remarkable lobular hepatitis could be regarded as the evidence of “VCZ- induced liver injury” (Figure 5 a-c). It should be noticed that given the high ALT levels (190 IU/L), our patient had received a much lower loading dose of VCZ (6 mg/kg/dose despite 9 mg/kg/dose) and also maintenance dose (4 mg/kg/dose despite 8 mg/kg/dose). Despite VCZ dose adjustment, both “VCZ-induced dermatologic toxicity” and liver injury were observed in this rare case. Therefore, exfoliative skin rash at the presence of high VCZ C _trough_ levels can be considered as a possible surrogate marker for detection of “VCZ-induced liver injury" in those patients with preexisting liver dysfunction”.

An association between VCZ C _trough_ level and specific side effects (e.g., CNS complications) has been documented previously. However, there is no evidence regarding the presence of such an association when it comes to complications, such as visual disturbances, liver injury, and skin rashes.

To the best of our knowledge, this is the first report that confirms an association between VCZ C _trough_ levels and dermatologic side effects. Similar studies can render helpful clues for the prediction of VCZ toxicity in the absence of state-of-the-art diagnostic genetic studies and even VCZ-TDM.

**Ethical considerations**

The present study was approved by the Ethics Committee of Prof. Alborzi Clinical Microbiology Research Center, Shiraz University of Medical Sciences. The research protocol conformed to the ethical guidelines of the 1975 Helsinki Declaration. In addition, written informed consent was obtained from the parents of the patient for the publication of this case report and any accompanying images.

## Conclusion

Generalized exfoliative skin rash should be considered a potential VCZ side effect that should be recognized and carefully managed early after treatment. When *CYP2C19* genotyping and VCZ-TDM are not available in the routine practice, attention to the early alarming signs of VCZ toxicity may be lifesaving to prevent severe VCZ toxicity, such as CNS side effects.
